# Heterogeneous Trajectories of Physical and Mental Health in Late Middle Age: Importance of Life-Course Socioeconomic Positions

**DOI:** 10.3390/ijerph14060582

**Published:** 2017-05-30

**Authors:** Eunsun Kwon, Sojung Park

**Affiliations:** 1 Center for Social Science, Seoul National University, 1 Gwanak-ro, Gwanak-gu, Seoul 08826, Korea; 2 George Warren Brown School of Social Work at Washington University in One Brookings Drive, Saint Louis, MO 63105, USA; spark30@wustl.edu

**Keywords:** cumulative disadvantage, life course, heterogeneous trajectories of physical and mental health, health disparities, late middle age

## Abstract

Drawing on life course and cumulative disadvantage theory, this study examines heterogeneous trajectories of functional limitations and depressive symptoms among late middle-aged individuals. This study used prospective data from 6010 adults, 51 to 64 years old, collected over a 12-year-period from the Health and Retirement Study. Considering the empirical proposition that several physical and mental trajectories may exist, Latent Class Growth Modeling was used. Five heterogeneous patterns of joint trajectories (*Relatively healthy*, *Moderately improving*, *Steadily deteriorating*, *Steeply deteriorating,* and *Persistently high comorbid*) were identified. Early life adversity was related to an increasing risk of declines in physical and mental health. The *Persistently high comorbid* class was characterized by a concentration of disadvantages over the life course. The development of public health interventions could help reduce co-existing physical and mental health problems, especially during late middle-age.

## 1. Introduction

Guided by a life course perspective, a growing body of research has suggested that early life socioeconomic disadvantages are significant predictors of mortality, morbidity, depression, functional limitations, and other health outcomes in later life [1]. Examination of the long-term effects of life-course factors on adult health is important since it can provide knowledge on how resources should be prioritized in designing policies and programs to address health disparities.

The present study aims to contribute to current life course research on adult health in three respects. First, the association between physical and mental health is well known [2,3]; co-existing physical and mental health problems have significant implications at an individual and societal level (e.g., increased number of work loss days) [4], premature mortality risk, and increased health service use [5]. Therefore, examining health problems separately in aging population is limiting because one condition often leads to the accumulation of simultaneous deficits due to the interrelationships [6].

Physical deficits tend to be accompanied by mental decline in older adults and these two terms, *physical* and *mental*, cannot be used interchangeably to identify the vulnerable subset of aging population requiring enhanced health care [7]. For example, depression is a risk factor for functional problems because it impairs cognitive capacity and affects health-related behaviors and psychological mechanisms. In contrast, functional limitations may contribute to depression as the degree of functional impairment changes physical appearance, which cause mental health problems or physical illness related to functional limitations may cause depression physiologically [8,9,10]. To improve development of effective and targeted strategies for prevention and treatment, it is important to examine differential longitudinal patterns of physical and mental health simultaneously [9]. Few research projects have examined both simultaneously with a focus on older adults [11]. Li et al. examined distinct courses of development of physical limitations and depressive symptoms over time and found significant correlations in the heterogeneous trajectories (e.g., old adults from the ‘Late Increase’ group in Instrumental Activities of Daily Living (IADL) scores tended to belong to the constantly high Center for Epidemiological Studies Depression Scale (CES-D) group), consistent with the findings of Yang and George [12,13]. Lenze et al. also found significant associations between persistently elevated depressive symptoms and a steep decline in functional disability [14]. Although these studies captured how the distinct trajectories of physical limitations are associated with those of depressive symptoms, they modeled the trajectory of a single health outcome at a time. To gain a better understanding of the distinct development of both health outcomes, a joint trajectory model is needed to identify heterogeneous groups of Americans experiencing different patterns of concomitant changes in both health domains.

Second, to date, little is known about the heterogeneity of health trajectories in middle age. Middle age is neither clearly defined nor well studied. It is vaguely described as the period between youth and old age [15], but most commonly considered to be ages from 40 to 60 [16]. Life course researchers have suggested the need to distinguish between early and late middle age based on the shift in allocation of resources over the life course. For example, early middle age is considered as the *ascendant phase* in which individuals are upwardly mobile both themselves and in relation to their families (e.g., achieving, helping children develop), while late middle age is considered the *executive phase* in which individuals reach maximum status in their social environments, which is followed by the *acceptant phase* that finds them beginning to lessen commitment to society and increasingly focusing on private pursuits [17]. In the last two phases of middle age, individuals tend to experience major life transitions such as career setbacks, changes in family status, and a decline in health status [18]. Exposure to socioeconomic disadvantages may be more deleterious to health in middle age because there are fewer income-generating years left compared to their younger counterparts. and most do not yet qualify for Social Security benefits (eligible at age 62) or Medicare (age 65) in the United States (U.S.) [19].

Previous studies have focused on late middle age (50s to early 60s) to obtain a better understanding of this age cohort [19,20]. For this study, we focus on this phase of life using the pre-retirement age cohort of the Health and Retirement Survey (HRS) aged 51 to 64. Most studies on heterogeneous trajectories of depressive symptoms or functional limitations have focused on either younger or older samples [21,22] or were based on individuals who were middle aged and older [23,24], which made it difficult to generalize the distinct patterns of depressive symptoms or functional health, or both, of middle-aged individuals. Across the adult age range, the average number of depressive symptoms was found to steeply decline in early middle age but to rise back to an intermediate level during late middle age [25,26]. The changes leading to functional limitations can also occur in late middle age as adults undergo various physical changes and physical activity declines [27], but, the value of physical and mental health in this life stage has not been clearly demonstrated [28].

Third, based on the notion of critical or sensitive periods, research has suggested that early life experiences make a direct and independent effect on adult health [29], but that socioeconomic conditions during other life stages contribute little [1]. For example, Galobardes, Lynch, and Davey Smith concluded that lower childhood socioeconomic status (SES) was associated with higher mortality rates among adults, regardless of socioeconomic circumstances during adulthood [30]. Ample evidence has also shown the robust association between childhood disadvantages and worse physical health in midlife after controlling for adult health behaviors and adult SES [5] or controlling for childhood health and adult SES [31]. Other research has argued that childhood factors do not have a direct impact on adult health [32,33] but an indirect influence through adulthood circumstances such as low educational attainment, low occupational status and low income, high unemployment, and even unstable marital status [34]. Childhood SES disadvantages appeared to affect later health through its effect on educational attainment and household income in adulthood [35], suggesting that the adverse effect of childhood disadvantages may be ameliorated if people from disadvantaged childhood achieve higher SES status in adulthood [31].

Little is known about whether the variations in meaning and importance of life course factors in old age apply to late middle age. However, most longitudinal research has not differentiated between health trajectories in late middle age and old age; their main focus has been on older adults [36]. Only a few studies have incorporated life course factors to examine both physical and mental health in middle age. Using structural equation modeling in a cross-sectional study, Laaksonen and colleagues examined the direct and indirect associations of various childhood circumstances and adulthood SES with physical and mental health among middle-aged individuals [37]. They found no direct relationship between childhood factors and either physical or mental health, but an indirect effect through adulthood SES. Interestingly, they found a divergent pattern of association: adulthood low SES was associated with poor physical health, but individuals with higher adulthood SES were more likely to have poorer mental health. In another cross-sectional study, Mäkinen and colleagues found that childhood adversities were associated with physical health problems later in life, but the association disappeared after adjusting for respondent’s education. In contrast, childhood adversities were related to mental health problems in adulthood, even after controlling for education [38]. This study was important because it showed that physical and mental health were differentially associated with childhood circumstances and suggested that education was a mediator for physical but not mental health. Although these limited studies provide important evidence on how different life course factors impact physical and mental health, none examined co-existing patterns of physical and mental health status over time.

This study has two objectives. First, we attempt to identify heterogeneous longitudinal patterns of functional limitation and depressive symptoms among late middle-aged adults over a 12-year observation period. Second, we explore to what extent the identified trajectories are associated with socioeconomic status over the life course. That is to say, we aim to examine whether childhood SES has an independent effect on the joint trajectories or whether adulthood SES is a mediator.

## 2. Materials and Methods

### 2.1. Data and Sample

Data were taken from seven waves of the HRS between 1998 and 2010 conducted by the University of Michigan in Ann Arbor with support from the National Institute of Aging. The HRS began in 1992 by surveying a nationally representative sample of more than 12,600 persons born between 1931 and 1941. In 1993, the Asset and Health Dynamics Among the Oldest Old (AHEAD) study was launched with a national sample of individuals born before 1924. We used 1998 as the baseline for our analysis because starting in 1998, the HRS and AHEAD surveys were fully integrated and two more subsamples were added in 1998: Children of the Depression Age (CODA: born between 1924 and 1930) and War Baby (WB: born between 1942 and 1947); both have been surveyed every two years since. At the time of our analysis, the HRS provided only an Early-Release version of 2012 data sets which did not include income and some health information, resulting in restricting the sample to individuals observed between 1998 and 2010.

For married and cohabiting respondents, HRS includes both partners in the sample. In order to reduce possible interdependence in estimating joint health trajectories, we restricted our sample to family respondents in each household, excluding 2756 non-family respondents (partners), which meant that our initial analytical sample consisted of 6712 middle-aged individuals aged between 51 and 64 between 1998 and 2010 (Observations = 25,887). Depressive symptoms are self-reported, so proxy respondents were not asked about these questions and were excluded. Those who never reported their childhood circumstances during the study period were also excluded. Childhood information was missing on poverty experience (0.18%), parental education (6.50%), and self-rated health (0.02%); adulthood information was missing for work status (0.02%) and educational attainment (0.14%). Preliminary analyses showed that respondents who were missing childhood information had similar outcomes in functional limitations and depressive symptoms. Since we built hierarchical models to examine the role of childhood and adulthood SES in the life course perspective, we compared the successive models. To use likelihood ratio tests to compare the models, listwise deletion was performed for missing information on the independent variables, which led to further reductions, resulting in a final sample of 6010 adults (observations = 24,008). Of the 6010 respondents, 338 (5.6%) responded at all seven waves, 679 (11.3%) for six, 816 (13.5%) for five, 1987 (33.0%) for four, 1537 (25.5%) for three, and 653 (10.8%) responded in two waves. Therefore, nearly 63.7% of the respondents completed at least four waves. In the final sample, 164 respondents died during the observation period (observations = 596) and 515 respondents missed at least one observation during the follow-up period for some reason other than death (observations = 1890). We included indicators of mortality (1 for those who died and 0 otherwise) and attrition (1 for respondents who missed at least one observation for some reasons other than death, and 0 otherwise) in the regression model.

Missing data are a problem with longitudinal surveys. The standard approach in life course research is to restrict analyses to individuals who have complete information for the key variables, mostly childhood variables, if the number of cases lost is not large and those respondents did not differ significantly on basic demographics from the original sample [31,35,39,40,41]. However, analysis results that excludes cases with missing data are valid only if the excluded respondent subsample is a completely random subset of the original sample and the data are missing completely at random, which is rarely true in population-based studies [42]. To check if missing data biased our findings, we performed supplemental analyses based on prior life course studies (results not shown but available upon request). First, a dummy variable adjustment for childhood variables (including parental education and childhood poverty experience) with substantial amounts of missing data was created and added to the regression model. Results with this dummy variable were not notably different from the results with the analyzed sample, similar to previous life course studies [43,44]. Second, preliminary analyses showed that respondents with missing childhood information had similar characteristics to those in the lower socio-demographic and socio-economic status (e.g., older, Black, with low education and income levels), consistent with prior findings using data from the HRS [45,46]. In supplemental analyses (not shown), including these low status missing cases produced similar results and left substantive conclusions unaffected. Lastly, we used Multiple Imputation (MI) to replace childhood variables with missing values with multiple (five in this study) simulated values [42] and results were similar to those with complete-case analysis in accordance with a number of previous studies [11,47,48].

### 2.2. Measures

*Mobility functional limitations.* We chose mobility functional limitation over Activities of Daily Living (ADL) and Instrumental Activities of Daily Living (IADL) limitations because less than 6% of middle-aged sample of the HRS had ADL/IADL limitations, which did not allow for enough variance for Latent Class Growth Modeling. As an alternative measure, we chose mobility functional limitations which are indicators of general physical ability [13,49] and are more appropriate for nonelderly populations, especially since 85% of the middle-aged sample of HRS had at least one limitation. Unlike ADL and IADL, mobility functional limitations measure access to specific physical activities, but are not associated with social roles [49,50]. Mobility tasks are indicators of general physical ability based on whether a respondent has difficulty performing particular physical tasks due to health problems. The tasks asked about were walking several blocks, jogging one mile, walking one block, sitting for two hours, getting up from a chair, climbing stairs, climbing one flight of stairs, stooping, reaching arms above shoulder level, pulling/pushing large objects, lifting or carrying weights over ten pounds, and picking up a dime. A score ranging from 0 to 12 was created by summing the problematic tasks. About 85% of the middle-aged sample was found to have at least one limitation during the observation period.

*Depressive symptoms.* The HRS depressive symptoms measure is a subset of the CES-D. The original CES-D contains 20 items designed to assess the level of depressive symptomatology; due to interview time constraints, HRS included an 8-item shortened version. Negative indicators are whether the respondent experienced the following all or most of the time: depression, everything is an effort, sleep is restless, felt alone, felt sad, and could not get going. Positive indicators measure whether the respondent felt happy and enjoyed life all or most of the time. A summary score (range: 0–8) was constructed with the positive items reverse coded such that a higher score indicated more negative symptoms.

*Ascribed factors.* Guided by the life course perspective, age, race, and gender were considered ascribed characteristics that individuals cannot change [51,52]. Age was measured in years at baseline, gender as female (1) and male (0), and race/ethnicity as Caucasians (0), African Americans (1), and others (2).

*Childhood socioeconomic circumstances.* In order to capture the overall level of socioeconomic standing of the family during childhood, parental education and family financial well-being were assessed. First, respondents were asked whether their family was financially well off, about average, or poor. We categorized respondents into those who said their family was poor (1) and others (0). Second, parental education was measured with a set of three dummy categories using the highest level of education completed by either mother or father: less than high school (0–11 years), high school graduate (reference group), and some college education and above (12+ years). The quality of retrospective childhood SES reports has been confirmed in a robust sociological literature [53], and these two variables from the HRS have been widely used [31].

*Adulthood socioeconomic circumstances.* Measures of adulthood SES include educational attainment and household income. Educational attainment was measured as a set of three dummy categories; those who did not finish high school were coded 1, those with college or higher education were coded 2, and those with high school diploma made up the reference group (coded 0). Due to high skewness, household income was log-transformed and categorized into ten groups for the deciles of logged income. Then, based on the change or stability of income, four categories were created: households whose income was maintained in the upper 60% of income deciles (reference; 0); households with increased income (increased; 1); those with decreased income (decreased; 2); and those whose income was constantly in the bottom 40% of income deciles (low-maintaining; 3) between the first and last observations.

*Covariates.* We created several dummies for respondents’ marital and work status based on change or stability. Marital status was measured as constantly married during the whole study period (0); constantly unmarried (1), and marital status changed (2). Work status was measured as constantly worked (0); constantly not working (1); and work status changed (2). Childhood health was measured using retrospective reports in which respondents were asked to assess their health before the age of 16 into five categories ranging from excellent (1) to poor (5). Our model also controlled for baseline health conditions: self-rated health, number of chronic diseases, and obesity. Respondents self-assessed their overall health ranging from excellent (1) to poor (5). Chronic diseases have been considered as important risk factor for depressive symptoms and treated as control variables [11,13]. Chronic diseases could be included in the latent class growth analysis (LCGA) model as a physical health outcome; however, an assumption of LCGA that all individual growth trajectories within a class are homogeneous did not seem to be the case for chronic diseases due to the existence of particular comorbid combinations. For example, individuals with the same number of chronic diseases could have different combinations, and the relative impacts across a range of comorbidities have been widely shown [54]. Therefore, we controlled for only baseline differences in the number of chronic diseases including high blood pressure, diabetes, cancer, lung disease, heart disease, stroke, and arthritis. We included obesity because it is considered to be one of the processes at work (functional limitations comorbid with depression) in this age group [55,56]. Body mass index (BMI) was calculated as weight in kilograms divided by the square of the height in meters and then, categorized into obesity (30.0 kg/m^2^ or higher) and not obesity (29.9 kg/m^2^ or lower).

### 2.3. Statistical Analyses

Considering the empirical proposition that several physical and mental trajectories may exist, Latent Class Growth Modeling (LCGM) was carried out using Mplus software (version 7.31, Muthén and Muthén, Los Angeles, CA, USA). This analytic technique identifies different latent trajectory classes, handling incomplete data on the health outcome variables by using all available observations to compute full information, maximum likelihood parameter estimates without imputing or dropping cases [57]. As very few respondents in our study were observed at all survey points, this feature was particularly useful.

The LCGM identifies heterogeneous classes by modeling a mixture of distinct multivariate normal distributions. The linear growth model provided a better fit with the data than a quadratic growth model and the function takes the following form:

(1) $$y_{it}^{j}=\;\alpha_{0}^{j}+\;\alpha_{1}^{j}\;Time_{it}+\;\varepsilon_{it}^{j}\;i=1,\ldots .n$$

where $y_{it}^{j}$ is a latent variable representing the depressive symptoms or mobility functional limitation of individual *i* at a given observation, *t* for group membership *j*. *Time* indicates number of years since the baseline. $\alpha_{0}^{j}$ and $\alpha_{1}^{j}$ are the coefficients associated with the intercept and slope of depressive symptoms or mobility functional limitation for individuals in group *j*. $\varepsilon_{it}^{j}$ represents a disturbance term assumed to be normally distributed with zero mean and constant variance. Like the approach to linear growth modeling for a continuous outcome, growth parameters of several health outcome trajectories can be used simultaneously, which contributes to the identification of latent classes [51]. The intercept and growth parameters from the individual class memberships for mobility functional limitation and depressive symptoms were used to predict the probability of multiple class membership in a joint statistical model. In pursuit of identifying joint group trajectory, we followed the analytical steps suggested in methodological and empirical literature [58,59]. This statistical model, which is an extension of the conventional LCGM, relates the entire longitudinal course of the two health outcomes and indicates the probability that the identified subclasses co-occur between the two [60]. For example, the co-occurring group is composed of people who share common growth parameters for both high mobility functional limitation and depressive symptoms. These conjoint latent classes may provide comorbidity of multiple health outcome trajectories in a longitudinal context and, thus, help us better understand the comorbidity of different forms of physical and mental health. Previous studies found that this dual modeling provides a more comprehensive understanding of the dynamic linkages between two longitudinal outcomes [59].

Then, we explored the characteristics of individuals within each class; the overall differences in percentages and standard errors were examined using χ^2^ tests and the overall differences in means were tested using Wald F-tests. Our final analytic task was to test whether latent class differences could be described by childhood and adulthood factors using a multinomial logistic regression approach. For these analyses, three models were estimated separately for testing the independent effects of life course factors in hierarchical models. Hierarchical models one through three present estimates of the effects of respondent-ascribed, childhood, and adulthood factors, respectively, on the joint trajectories. Each of these models further includes covariates. The successive models were compared based on the model fit (∆χ^2^ and pseudo-R^2^). Although our ‘most likely class membership’ approach yielded high entropy (0.80), it should be noted that this approach can have a potential underestimation of the standard errors of the parameter [61]. Therefore, when evaluating the role of predictor variables, we employed a more stringent criterion than the 5% level (*p* < 0.01) for deciding on significance of variables. For analyses, STATA (version SE 13.1, StataCorp LLC, College Station, Texas, USA) was used throughout.

## 3. Results

### 3.1. Trajectories of Mobility Functional Limitation and Depressive Symptoms

The LCGM modeling approach was used to identify trajectories of mobility functional limitations and depressive symptoms among middle-aged adults. We established the optimal number of latent classes to identify different trajectory classes. Models one through eight classes were tested and evaluated using the following five common indices: (a) Bayesian information criterion (BIC; [62]), (b) sample-sized adjusted BIC (SSABIC; [63]), (c) Entropy Index [64], (d) Lo-Mendell-Rubin (LMR) likelihood ratio test of model fit [65], and (e) bootstrapped likelihood ratio test [66]. Lower values of BIC and SSABIC indicate a better fitting model [60] and significant BLRT and LMR χ^2^ values (*p* < 0.05) indicate that the specified model fits the data better than the specified model with other classes [64]. Given the large sample size, which affects values of Akaike information criterion (AIC) and BIC, we considered three more criteria: class interpretability (whether an additional class provided unique information), class prevalence (we defined adequate class size as at least 1% of the sample) [58], and high posterior probabilities (near 1.0). The 6-class model and the model with more classes (results not shown) showed a small improvement in model fit (i.e., lower values of BIC and SSABIC), but resulted in smaller and less interpretable classes (less than 1%) (Table 1). Thus, the five-class model was chosen.

**Figure 1:**
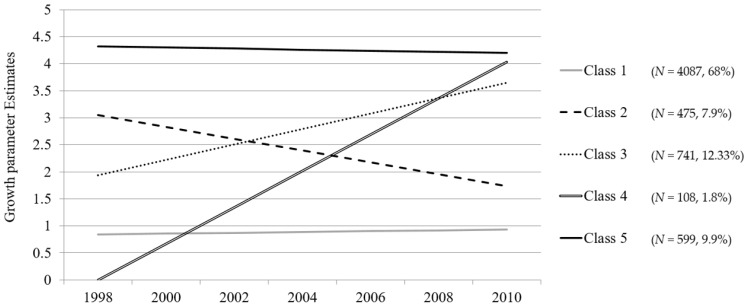
Estimated linear latent growth curves from joint health trajectory modeling.

Figure 1 reflects the intercept and growth parameter estimates from joint trajectory modeling with mobility functional limitations and CES-D. The model-based parameter estimates from the growth model indicated that across the identified five classes there were significant initial differences in both the health trajectories and individual differences in the rate of change (slope) across the seven waves of biannual assessment. Specifically, five distinct patterns were described by growth curve estimates with different intercepts and slopes. Individuals in Class 1 were in relatively good health and had linear rate decreases on average by 0.015, indicating relatively minimal declines. Those in Class 2 had poorer health at baseline than Class 1 (3.049) but experienced improvement by 0.219 for each time point. The opposite trend was observed for Class 3, with better health at baseline (1.936) than Class 2 but a steady decline by 0.285. Those in Class 4 started with the best relative health status at baseline (0.000), but experienced steep deteriorating health over time by 0.672, which was the second poorest health at the final time point (4.032). Individuals in Class 5 had persistently poorer health relative to those in the other four classes.

**Figure 2:**
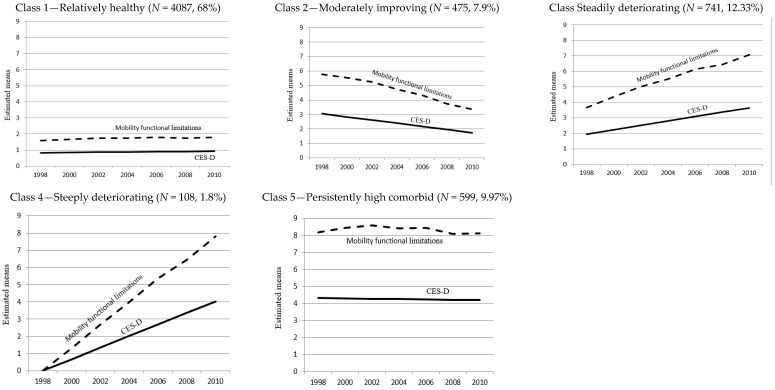
Joint trajectories of mobility functional limitation and Center for Epidemiologic Studies Depression Scale (CES-D).

Figure 2 illustrates the observed joint trajectories in each class. Among the five trajectories, *Relatively healthy* (Class 1) and *Persistently high comorbid* (Class 5) showed a fairly stable pattern during the observed study period. Respondents in *Relatively healthy* (Class 1) experienced very low mobility functional limitations and depressive symptoms over time, while the opposite was true for the *Persistently high comorbid* group (Class 5). The other three trajectory groups showed dynamic patterns of change: Mobility functional limitations and depressive symptoms decreased in *Moderately improving* (Class 2) while they increased in *Steadily deteriorating* (Class 3). Both health outcomes became substantially worse in *Steeply deteriorating* (Class 4).

### 3.2. Characteristics of Trajectory Groups by Childhood and Adulthood Factors

Table 2 presents results comparing ascribed, childhood, and adulthood characteristics across the identified trajectory groups. There were significant differences for all the variables across the trajectory classes. The *Relatively healthy* group (Class 1) was the most prevalent trajectory group (68% of the sample); individuals in this group had the fewest childhood disadvantages compared with the other groups: they were less likely to have financial difficulties, parents with low education, and poor health during childhood. In adulthood, they had the highest proportion of people with high education, consistently higher income with some experiencing an increased income, and stable marital and work status.

In contrast, the *Persistently high comorbid* group (Class 5) was characterized by a high concentration of disadvantages over the life course: poverty, parents with low education, and poorest self-rated health in childhood; in adulthood, they had the lowest level of education and consistently low income. They also tended be constantly unmarried and unemployed throughout the study period and to have highest number of chronic conditions. *Moderately improving* (Class 2), *Steadily deteriorating* (Class 3), and *Steeply deteriorating* (Class 4) groups reported relatively less disadvantageous attributes than the *Persistently high comorbid* group, but more disadvantaged than *Relatively healthy* (Class 1).

### 3.3. Role of Childhood and Adulthood SES in Predicting Trajectory Membership

Multinomial logistic regression was used to assess how ascribed, childhood, and adulthood factors affected the probability of membership in latent classes (Table 3). All relative risk ratios (RRR) are compared to the likelihood of membership in the *Relative healthy* group. In Model 1 with ascribed factors only, compared with the *Relatively healthy* group (Class 1), those who were older were more likely to be in the *Moderately improving* (Class 2) or *Persistently high comorbid* (Class 5) group while younger individuals were more likely to be in the *Steeply deteriorating* group (Class 4). Females were more likely to be in *Moderately improving, Steadily deteriorating,* and *Persistently high comorbid* groups than in the *Relatively healthy* group, and those in racial groups other than Caucasians tended to be in the other four classes rather than in the *Relatively healthy* group. In Model 2, childhood factors were added and provided a slightly better model fit (comparison of ∆χ^2^(16) = 2149.15, *p* < 0.001 and of ∆ pseudo-R²). Individuals who experienced childhood poverty and had parents with less than a high school education were more likely to be in the *Steadily deteriorating, Steeply deteriorating,* or *Persistently high comorbid* group than the *Relatively healthy* group. The likelihood of being in the *Moderately improving* group was positively associated with low parental education. Those with poor childhood self-rated health were less likely to be in the *Relatively healthy* group.

In Model 3, adulthood factors were added and provided a substantively improved model fit (comparison of ∆χ^2^(48) = 11,093.00, *p* < 0.001 and of ∆ pseudo-R^2^). The small effects of childhood factors remained significant but diminished after adulthood factors were included, suggesting that the addition of adulthood factors added to predicting group membership. Among adulthood factors, increasing, decreasing, and low maintaining incomes were associated with a likelihood of being in the *Steadily deteriorating, Steeply deteriorating,* and *Persistently high comorbid* groups, while those with decreasing or low maintaining incomes were more likely to belong to the *Moderately improving* group. Low education of respondents was found in the *Steeply deteriorating* and *Persistently high comorbid* groups. Those who were constantly unmarried or unemployed were more likely to belong to any of the four groups except *Relatively healthy*. Especially the effects of their work status appeared to be large for the *Persistently high comorbid* group. Respondents’ baseline health conditions were also significantly associated with group placement: those with poorer self-rated health and a higher number of chronic conditions were less likely to be in the *Relatively healthy* group, and those who were obese were most likely to be in the *Moderately improving, Steadily deteriorating,* or *Persistently high comorbid* groups.

## 4. Discussion

To the best of our knowledge, this study is the first to examine heterogeneous trajectories of functional limitations and depressive symptoms among late middle-aged individuals. We believe that the joint trajectories may have captured the comorbidity of different health problem trajectories in a longitudinal context and thus reflect a stronger association with life experiences compared with latent classes of specific health outcomes. Our analytical technique also captured intra-individual change in health, which is important because individuals are heterogeneous in terms of the rate of change in health [51].

Changes in health status are characterized by many biological and psychological changes [67]; comprehensive assessment of physical and mental health domains helps us better understand how the trajectories of one domain are linked to those of the other domains, as well as identify distinct groups of joint trajectories across multiple health outcomes [11]. The trajectory model of a single health outcome cannot achieve these findings, but provide key evidence that there are heterogeneous trajectories for each outcome as well as correlations between the two outcomes when examining separate group-based models of a single health outcome [12]. However, researchers and clinicians still need to explore whether depression treatment helps physical functioning over time or vice versa. If the two health outcomes change in the same direction, an intervention could be targeted at those with health decline; however, if the two outcomes go in opposite directions for specific groups, group-specific interventions could be considered. This study has contributed by introducing and illustrating a joint modeling method designed for studying comorbidity in dynamic and yet more realistic ways.

Our findings make several important contributions to the life course research on adult health. First, from the dual trajectories we observed there was a fair amount of overlap of movement across most trajectory groups between mobility functional limitations and depressive symptoms in late middle age. Changes in physical and mental health were not homogeneous, but diverged into five different patterns. Two contrasting groups, *Relatively healthy* and *Persistently high comorbid*, showed stability over time, while three groups, *Moderately improving*, *Steadily deteriorating*, and *Steeply deteriorating*, exhibited dynamic patterns of change. 

The *Persistently high comorbid* and *Steeply deteriorating* groups merit particular attention. The findings suggest that those who experience both physical and mental health problems are exposed to the risk of co-morbidity, although it was not our goal to assess clinical morbidity. The standard cut-off score for depression is four [68] and for functional limitations is one [50]. Individuals in the *Persistently high comorbid* group exhibited higher levels of co-morbidity, with more than eight mobility functional limitations out of twelve and constant depressive symptoms above the score of four over the 12-year study period. Those in the *Steeply deteriorating* group had an important change pattern: They were more likely to have no impairment in either outcome at the baseline, but this changed over the study period. In order to further examine morbidity in the *Persistently high comorbid* and *Steeply deteriorating* groups, auxiliary analyses were examined with additional indicators of physical health, including ADL skills which are commonly used as an indicator of disability. The results showed that individuals in the *Persistently high comorbid* group had more than two ADLs, suggesting they are at high risk for disability.

Second, in this study life course factors were significantly and differentially associated with each class. The two stable groups, *Persistently high comorbid* and *Relatively healthy*, showed contrasting patterns of association with life course factors: consistent with our expectations, there was evidence of a significant influence of socioeconomic disadvantages on the *Persistently high comorbid* class, which was characterized by a concentration of disadvantages including low childhood and adulthood SES along with unstable or poor marital and work status. Both childhood poverty and low parental education were significantly associated with membership in the *Steadily deteriorating*, *Steeply deteriorating*, or *Persistently high comorbid* group. Adjusting for adulthood factors, the effect sizes decreased but still remained significant. Especially, the factors associated with being in the *Steadily deteriorating* or *Persistently high comorbid* group are consistent with the characteristics of multidimensional health problems identified in previous studies [11,51]. For example, Wickrama and colleagues found that lower childhood socioeconomic conditions, education, and income were associated with joint trajectories featuring greater impairment or more deterioration in multidimensional health outcomes [51]. Our findings add to these studies by providing new evidence that early life adversity is related to an increasing risk of declines in physical and mental health in late middle age, supporting the critical period perspective that argues for persistent negative effects of early life disadvantages on adult health. It is also noted that, in our sample, about 26% of people from disadvantaged childhood had low education and 18% of those had low maintaining incomes, suggesting the possibility of increasing the risk of health problems through gradually accumulated exposures to socioeconomic disadvantages over the life course. Future studies may need to speculate which protective factors would break the chain of risk for health problems for these people with cumulative disadvantages. For example, more educational opportunities may buffer the adverse effects of childhood disadvantages and cause less intergenerational dependence by promoting their self-reliance and social competence [69].

One of strengths in our study is that we explored a range of change/stability patterns of adulthood factors. We found significant impacts for both childhood poverty and low-maintaining income in adulthood on the likelihood of being in the *Steadily deteriorating*, *Steeply deteriorating*, and *Persistently high comorbid* groups. Our measurement of income change was based on movement of ten percentiles of income distribution, which requires large absolute changes in income for individuals. Our findings demonstrate that childhood and adulthood factors are not mutually exclusive; the examination of change/stability patterns in adulthood factors provides important implications for future research as only few studies have demonstrated the influence of socioeconomic changes on adult health [70].

A few interesting findings emerged when comparing the groups. Compared the *Steeply deteriorating* group with the *Steadily deteriorating* group, we found that low parental education was more likely to be a factor in the steady decline in physical and mental health (*Steadily deteriorating* class) although the effect size was modest, while respondents’ low education mattered for the steep decline (*Steeply deteriorating* class). For the *Steeply deteriorating* group, unstable work and income statuses appeared to have greater influences than education: In additional analyses, we observed that 56.45% of that group was unskilled or low-wage service workers, which was a relatively higher proportion than for other groups (42% on average). However, this may be associated with low education which can significantly affect the likelihood of experiencing a substantial decline in health, especially the steep increases in mobility functional limitations observed in Figure 2. Negative socioeconomic changes were associated with low education, including decreasing income (50%), which might have contributed to the decline in physical health through emotional and psychosocial attributes or lack of resources/social support [71]. This lends support to the theoretical and empirical hypothesis that stressful conditions contribute to the risk of functional disability, which was found to be the first observable evidence of depressive symptoms [13,72]. Previous studies have shown that functional disability produces effects quickly on depressive symptoms, but that depressive symptoms have a delayed effect on physical health [73]. It seems plausible that the steep decline in functional ability may lead to an increase in depressive symptoms and an increase in the reciprocal effects between the two health outcomes, but further research is needed.

For those in the *Steadily deteriorating* class, low education did not predict the likelihood of decline in functional ability, but low parental education did. Although the effect size was small, the continued effect after adjusting for all the adulthood factors supports previous findings that parent’s education *per se* reflects a range of noneconomic social characteristics such as general and health-related knowledge, literacy, social networks, and problem-solving skills, which are often perceived as enhancing a person’s efficacy in promoting health [32,45]. Physical and mental health appeared to change at similar rates in the opposite direction in individuals in the *Steadily deteriorating* (slope −0.22) and *Moderately improving* (slope +0.28) groups. Various protective factors may have contributed to the improvement in health status in these two groups (e.g., positive environmental factors in terms of social support) [74,75]. The examination of possible protective factors would be an important inquiry for future research.

In future research, exploring the relationship between earlier life and later life based on these findings could help to illuminate the importance of late middle age. Individuals’ experiences in childhood can affect health in late middle age, which, in turn, can have long-term consequences for later life. At the same time, there is still some opportunity for building resilience in this phase [16]. Individuals in late middle age are at an intersection requiring an optimal balance of growth (e.g., knowledge, experience) and declines (e.g., health); the opportunity to modify circumstances may be critical [17]. Future research should perform more detailed assessments of health changes across the whole lifespan in the life course perspective. For example, for similar distinct trajectories of depressive symptoms (*increasing*, *decreasing*, *high maintaining*, and *low maintaining*) have already been found across age cohorts including limited findings on late middle aged adults [41,76]; the largest class was characterized by persistently low or minimal depressive symptoms. However, the proportion of the sample in chronically high depressive symptom trajectory groups seemed to decrease with age [77]. Not only the examination of heterogeneous trajectories over the life course but of predicting factors for the distinct trajectories would expand our understanding of the lifelong effects of SES and depression.

We also note some limitations in this study. First, the association between income changes and the *Persistently high comorbid* and *Steeply deteriorating* classes is hard to explain because there is a possibility of reverse causality. It may be that those whose income was constantly low or decreased tended to experience constant health problems or worsening health status because of their disadvantaged economic conditions; however, it could also be that those with constant health problems or worsening health status were unable to work, resulting in decreased income or low maintaining income. Second, the small number of cases in the *Steeply deteriorating* group might have contributed to the limited effects of life course factors. Although a similar number of cases were used for distinct class membership in prior studies [58], the findings need to be interpreted with caution. Third, the examination of distinct patterns in the multi-dimensional trajectories of physical, emotional, and cognitive functioning in relation to socioeconomic stratification allows us to gain a better understanding of how this is an interrelated process [11,51]. In this study, we intended to consider cognitive functioning as a main health outcome and include it in the LCGA model. However, an age screen was used in HRS starting in 1998 to determine which cognition measures would be administered; specifically, all respondents 65 years of age and older received the full set of performance tests previously asked only in the AHEAD study, while respondents under age 65 were asked only to assess their memory at present compared with the way it was at the last interview and were not routinely asked of the question [11], which ultimately made it inappropriate for our analytic modeling. Considering the contribution of socioeconomic position over the life course to differences in cognitive decline [78], future research should include cognitive functioning as an additional domain, which would expand our understanding of health trajectories in late middle age.

## 5. Conclusions

Despite these limitations, our study identified significant heterogeneity in the joint trajectories of physical and mental outcomes with five differential patterns of longitudinal changes. The findings also extended existing literature by providing evidence of the association between socioeconomic disadvantages over the life course and joint health trajectories. This supports the need for the development of public health interventions that may help reduce co-existing physical and mental health problems, especially during late middle-age to provide a more comprehensive approach integrating medical care with psychosocial interventions, non-medical interventions which may be particularly beneficial for those with high rates of comorbidity.

## Figures and Tables

**Table 1:** Fit statistics for CES-D and Mobility Functional Limitation in middle-aged population (1998–2010).

Number of Classes	Log Likelihood	AIC	BIC	SA-BIC	Entropy	LRT *p* Value	Class Proportions	
1	−109,191.729	218,433.458	218,602.897	218,523.453	a	a		
2	−106,864.897	213,785.794	213,975.566	213,886.589	0.891	0.0000	Class 1: 5709 (95.5%)	Class 2: 301 (4.5%)
3	−105,982.392	212,026.785	212,236.889	212,138.379	0.853	0.0000	Class 1: 4531 (75.4%) Class 2: 1189 (19.8%)	Class 3: 290 (4.8%)
4	−105,776.791	211,621.583	211,852.020	211,743.976	0.828	0.0000	Class 1: 3966 (66.3%) Class 2: 1142(19.4%)	Class 3: 240 (3.1%) Class 4: 661 (11.2%)
5	−105,584.119	211,242.238	211,493.008	211,375.431	0.799	0.0084	Class 1: 4087 (68%) Class 2: 475 (7.9%) Class 3: 741 (12.3%)	Class 4: 108 (1.8%) Class 5: 599 (9.9%)
6	−105,471.078	211,022.156	211,293.258	211,166.148	0.788	0.0051	Class 1: 3546 (58.8%) Class 2: 541 (9.3%) Class 3: 300 (4.5%)	Class 4: 901 (15.5%) Class 5: 60 (0.9%) Class 6: 662 (11%)

Note: CES-D: Center for Epidemiologic Studies Depression Scale; AIC: Akaike information criterion; BIC: Bayesian information criterion; SA-BIC: Sample-size-adjusted BIC; LRT: likelihood ratio test; a: not estimable for a 1-class model.

**Table 2:** Baseline characteristics of trajectory groups (mean (standard deviation), or *n* (%)).

		Total	Class 1	Class 2	Class 3	Class 4	Class 5	Statistics
		Sample	Relatively Healthy	Moderately Improving	Steadily Deteriorating	Steeply Deteriorating	Persistently High Comorbid	
		*n* = 6010	*n* = 4087 (68%)	*n *= 475 (7.9%)	*n* = 741 (12.33%)	*n* = 108 (1.8%)	*n *= 599 (9.97%)	
Ascribed factor	Age	55.05 (3.13)	54.99 (3.17)	55.46 (3.02)	54.97 (3.04)	53.81 (2.31)	55.50 (3.08)	F(6010) = 11.20 ***
	Race							$x^{2}$(8) = 106.31 ***
	Caucasian	4635 (77.12)	3296 (80.65)	339 (71.37)	537 (72.47)	75 (69.45)	388 (64.77)	
	African American	1016 (16.91)	580 (14.19)	100 (21.05)	158 (21.32)	21 (19.44)	157 (26.21)	
	Others	359 (5.97)	211 (5.16)	36 (7.58)	46 (6.21)	12 (11.11)	54 (9.02)	
	Female	3545 (58.98)	2288 (55.98)	271 (57.05)	511 (68.96)	57 (52.77)	418 (69.78)	$x^{2}$(4) = 153.18 ***
Childhood factor	Poverty	1608 (26.76)	923 (22.58)	145 (30.53)	246 (33.20)	41 (37.96)	253 (42.24)	$x^{2}$(4) = 135.62 ***
	Self-rated health^ a^	1.95 (0.01)	1.77 (0.94)	2.26 (1.13)	2.15 (1.08)	2.22 (1.14)	2.63 (1.23)	F(6010) = 119.33***
	Parental education							$x^{2}$(8) = 206.82 ***
	Less than HS	1181 (19.65)	895 (21.90)	72 (15.15)	118 (15.92)	16 (14.82)	80 (13.36)	
	HS graduates	3498 (58.20)	2132 (52.16)	336 (70.73)	504 (68.02)	71 (65.74)	455 (95.96)	
	College or higher	1331 (22.15)	1060 (25.94)	67 (14.11)	119 (16.06)	21 (19.44)	64 (10.68)	
Adulthood factor	Marital status ^b^							$x^{2}$(8) = 146.66 ***
	Constantly married	3178 (52.88)	2349 (57.47)	229 (48.21)	341 (46.02)	48 (44.44)	211 (35.22)	
	Constantly unmarried	2014 (33.51)	1197 (29.29)	185 (38.95)	291 (39.27)	42 (38.89)	299 (49.92)	
	Status changed	818 (13.61)	541 (13.24)	61 (12.84)	109 (14.71)	18 (16.67)	89 (14.86)	
	Work status ^b^							$x^{2}$(8) = 1.10 ***
	Constantly worked	2428 (40.40)	2057 (50.33)	134 (28.21)	179 (24.16)	20 (18.52)	38 (6.34)	
	Never worked	1463 (24.34)	599 (14.66)	176 (37.05)	243 (32.79)	26 (24.07)	419 (69.95)	
	Status changed	2119 (35.26)	1431 (35.01)	165 (34.74)	319 (43.05)	62 (57.41)	142 (23.71)	
	Education							$x^{2}$(8) = 593.42 ***
	Less than HS	1075 (17.89)	463 (11.33)	123 (25.89)	182 (24.56)	31 (28.70)	276 (46.08)	
	HS graduates	2051 (34.12)	1349 (33.01)	185 (38.95)	304 (41.03)	34 (31.48)	179 (29.88)	
	College or higher	2884 (47.99)	2275 (55.66)	167 (35.16)	255 (34.41)	43 (39.81)	144 (24.04)	
	Monthly income ($)	5883.11 (8665.44)	7050.52 (11,406.2)	4143.79 (7127.3)	3835.93 (3947.54)	4111.71 (5789.76)	2148.95 (3426.09)	F(6010) = 48.86 ***
	Logged income	10.47 (0.02)	10.77 (1.48)	10.06 (1.84)	10.13 (1.69)	9.94 (2.29)	9.31 (2.02)	F(6010) = 130.97 ***
	Income change ^b^							(12) = 534.62 ***
	High maintaining Increased	765 (12.73) 2156 (35.87)	654 (16.00) 1549 (37.90)	40 (8.42) 158 (33.26)	55 (7.42) 248 (33.47)	4 (3.70) 32 (29.63)	12 (2.00) 169 (28.21)	
	Decreased	2206 (36.71)	1516 (37.09)	174 (36.63)	283 (38.19)	54 (50.00)	179 (29.88)	
	Low maintaining	883 (14.69)	368 (9.00)	103 (21.68)	155 (20.92)	18(16.67)	239 (39.90)	
	Health status							
	CES-D	1.71 (2.11)	1.04 (1.52)	3.06 (2.38)	2.27 (2.09)	1.31 (1.55)	4.59 (2.36)	F(6010) = 629.17 ***
	Functional limitation	2.88 (2.76)	1.51 (1.36)	6.02 (1.77)	4.23 (2.04)	1.46 (1.31)	8.35 (1.72)	F(6010) = 3390.53 ***
	N of chronic diseases	1.24 (1.23)	0.86 (0.94)	1.74 (1.22)	1.81 (1.19)	1.25 (1.06)	2.76 (1.50)	F(6010) = 515.14 ***
	Obesity (*N*, %)	2057 (34.23)	1121 (27.43)	200 (42.11)	370 (49.93)	35 (32.41)	331 (55.26)	$x^{2}$(4) = 296.05 ***
	Self-rated health	2.68 (1.15)	2.25 (0.93)	3.48 (0.96)	3.26 (0.97)	2.81 (1.02)	4.28 (0.81)	F(6010) = 826.19 ***

^a^ higher values indicate poorer health; ^b^ the category variables were created based on change and stability observed during the whole observation period, significance level of *p-*value *** *p* < 0.001; HS: High school.

**Table 3:** Multinomial logistic regression model predicting trajectory membership.

	Model 1: Ascribed Factors								Model 2: + Childhood Factors								Model 3: + Adulthood Factors							
	Class 2: Moderately Improving		Class 3: Steadily Deteriorating		Class 4: Steeply Deteriorating		Class 5: Persistently High comorbid		Class 2: Moderately Improving		Class 3: Steadily Deteriorating		Class 4: Steeply Deteriorating		Class 5: Persistently High comorbid		Class 2: Moderately Improving		Class 3: Steadily Deteriorating		Class 4: Steeply Deteriorating		Class 5: Persistently High Comorbid	
	RRR (SE)	*p*	RRR (SE)	*p*	RRR (SE)	*p*	RRR (SE)	*p*	RRR (SE)	*p*	RRR (SE)	*p*	RRR (SE)	*p*	RRR (SE)	*p*	RRR (SE)	*p*	RRR (SE)	*p*	RRR (SE)	*p*	RRR (SE)	*p*
**Person-ascribed**																								
Age	0.96	0.000	1.01	0.044	.96	0.008	1.02	0.000	1.03	0.000	1.00	0.524	0.96	0.002	1.01	0.013	1.02	0.000	0.98	0.059	0.94	0.000	0.99	0.466
	(0.01)		(0.00)		(0.01)		(0.00)		(0.00)		(0.00)		(0.01)		(0.00)		(0.00)		(0.00)		(0.01)		(0.00)	
Female	0.98	0.000	1.55	0.000	0.98	0.902	1.77	0.000	1.71	0.000	1.54	0.000	0.99	0.964	1.76	0.000	2.49	0.000	2.07	0.000	1.11	0.369	3.90	0.000
	(0.11)		(0.08)		(0.11)		(0.11)		(0.12)		(0.08)		(0.11)		(0.11)		(0.19)		(0.13)		(0.13)		(0.36)	
Race (Caucasian)																								
African American	1.52	0.000	1.66	0.000	1.52	0.001	2.46	0.000	1.41	0.000	1.40	0.000	1.25	0.062	1.85	0.000	0.86	0.041	0.81	0.001	0.96	0.808	0.73	0.000
	(0.18)		(0.08)		(0.18)		(0.13)		(0.08)		(0.07)		(0.15)		(0.10)		(0.06)		(0.04)		(0.12)		(0.05)	
Others	2.39	0.000	1.52	0.000	2.39	0.000	2.43	0.000	1.50	0.000	1.21	0.027	1.85	0.000	1.61	0.000	1.00	0.967	0.87	0.187	1.50	0.015	0.73	0.012
	(0.38)		(0.12)		(0.38)		(0.020)		(0.14)		(0.10)		(0.30)		(0.07)		(0.11)		(0.08)		(0.25)		(0.09)	
**Childhood SES**																								
Poverty									1.03	0.492	1.30	0.000	1.70	0.000	1.49	0.000	0.99	0.939	1.23	0.000	1.57	0.000	1.24	0.001
									(0.05)		(0.05)		(0.17)		(0.07)		(0.06)		(0.06)		(0.16)		(0.08)	
Parental education																								
<High school									1.57	0.000	1.56	0.000	1.53	0.002	1.86	0.000	1.25	0.003	1.24	0.000	1.31	0.049	1.28	0.007
									(0.10)		(0.08)		(0.20)		(0.12)		(0.09)		(0.07)		(0.18)		(0.12)	
College or higher									0.72	0.000	0.91	0.197	1.23	0.182	0.73	0.001	0.86	0.126	1.16	0.050	1.54	0.007	1.06	0.611
									(0.06)		(0.06)		(0.19)		(0.06)		(0.08)		(0.08)		(0.025)		(0.12)	
**Adulthood SES**																								
Income change ^b^																								
Increased																	1.27	0.016	1.41	0.000	2.42	0.000	2.55	0.000
																	(0.12)		(0.12)		(0.16)		(0.42)	
Decreased																	1.33	0.004	1.41	0.000	3.54	0.000	2.41	0.000
																	(0.13)		(0.12)		(0.88)		(0.40)	
Low maintaining																	1.70	0.000	2.16	0.000	2.98	0.000	3.91	0.000
																	(0.19)	(0.21)			(0.82)		(0.68)	
Education ^a^																								
<High school																	1.07	0.356	1.04	0.475	1.66	0.000	1.47	0.000
																	(0.07)		(0.06)		(0212)		(0.11)	
College or higher																	0.81	0.002	0.64	0.000	0.83	0.114	0.70	0.000
																	(0.05)		(0.03)		(0.09)		(0.05)	
**Covariates**																								
Marital status																								
Constantly unmarried																	1.41	0.000	1.56	0.000	1.55	0.000	2.08	0.000
																	(0.08)		(0.08)		(0.17)		(0.15)	
Status changed																	1.22	0.021	1.28	0.001	1.70	0.000	1.55	0.000
																	(0.10)		(0.09)		(0.23)		(0.16)	
Work status																								
Constantly not work																	2.12	0.000	2.959	0.000	3.28	0.000	11.87	0.000
																	(0.15)		(0.16)		(0.49)		(1.29)	
Status changed																	1.33	0.000	2.23	0.000	3.54	0.000	4.24	0.000
																	(0.08)		(0.12)		(0.44)		(0.46)	
Childhood health ^c^									1.53	0.000	1.36	0.000	1.41	0.000	1.93	0.000	1.15	0.000	1.06	0.007	1.20	0.000	1.23	0.000
									(0.03)		(0.02)		(0.05)		(0.03)		(0.02)		(0.02)		(0.05)		(0.03)	
(Baseline) Adult health																								
Self-rated health ^c^																	2.72	0.000	2.03	0.000	1.28	0.000	5.34	0.000
																	(0.08)		(0.05)		(0.06)		(0228)	
Number of chronic diseases																	1.49	0.000	1.60	0.000	1.29	0.000	2.02	0.000
																	(0.03)		(0.03)		(0.06)		(0.05)	
Obesity																	1.36	0.000	1.91	0.000	1.06	0.515	2.18	0.000
																	(0.07)		(0.08)		(0.11)		(0.14)	
Died during follow-up	2.77	0.001	4.25	0.000	5.41	0.000	7.44	0.000	3.02	0.000	4.56	0.000	5.91	0.000	8.72	0.000	1.61	0.150	2.62	0.000	4.17	0.001	2.50	0.003
	(0.81)		(0.95)		(2.22)		(1.56)		(0.89)		(1.04)		(2.44)		(1.92)		(0.54)		(0.70)		(1.77)		(0.77)	
Ever attrited	1.08	0.390	1.08	0.280	0.89	0.560	1.07	0.384	1.07	0.457	1.07	0.367	0.89	0.539	1.07	0.406	1.01	0.857	0.99	0.976	0.79	0.240	0.99	0.972
	(0.09)		(0.08)		(0.16)		(0.09)		(0.09)		(0.08)		(0.16)		(0.09)		(0.10)		(0.08)		(0159)		(0.11)	
**Constant**	0.00	0.000	0.03	0.000	0.18	0.027	0.00	0.000	0.00	0.000	0.02	0.000		0.002	0.00	0.000	0.00	0.000	0.00	0.000	0.01	0.000	0.00	0.000
	(0.00)		(0.01)		(0.14)		(0.00)		(0.00)		(0.00)				(0.00)		(0.00)		(0.00)		(0.01)		(0.00)	
Pseudo R^2^	0.0160								0.0592								0.2818							
Log Likelihood chi^2^	797.69 (24) ***								2946.84 (40) ***								14,039.84(88) ***							
(df)									2149.15 (16) ***								11,093.00(48) ***							
∆χ^2^																								

Note: SES: Socioeconomic status; reference group: *Relatively healthy* (Class 1); RRR: Relative Risk Ratios; SE: Standard Errors; ^a^ the reference group for parental education and respondent’s education is high school graduates; <High school indicates less than high school diploma; ^b^ the reference group for income change is those with constantly high maintaining incomes; ^c^ higher values indicate poorer health; significance level of *p-* value *** *p* < 0.001.
